# Associations between Personality Traits and Areas of Job Satisfaction: Pay, Work Itself, Security, and Hours Worked

**DOI:** 10.3390/bs13060445

**Published:** 2023-05-26

**Authors:** Weixi Kang, Antonio Malvaso

**Affiliations:** 1 Department of Brain Sciences, Imperial College London, London W12 0BZ, UK; 2 Department of Brain and Behavioral Sciences, University of Pavia, 27100 Pavia, Italy

**Keywords:** personality, Big Five, job satisfaction, pay satisfaction

## Abstract

Although studies have widely explored the connections between personality traits and job satisfaction, less is known about how personality relates to aspects of job satisfaction. The objective of this study was to explore the relationships between personality traits and various areas of job satisfaction, including pay, work, security, and hours worked. This study used ordinal regressions to analyze data from 6962 working individuals from the British Household Panel Survey (BHPS). The results showed that Neuroticism consistently has a negative association with all aspects of job satisfaction, whereas Agreeableness and Conscientiousness have positive associations with job satisfaction. Extraversion had a weak negative association with satisfaction with total pay. These findings imply that personality may play a crucial role in shaping areas of job satisfaction.

## 1. Introduction

The Five-Factor Model of Personality, commonly referred to as the Big Five, is comprised of five dimensions: Agreeableness, Extraversion, Conscientiousness, Openness, and Neuroticism [1]. Extraversion is characterized by assertiveness and sociability, whereas Agreeableness is associated with politeness and cooperativeness. Conscientious individuals are known for their organizational skills and task-oriented focus, while those high in Neuroticism are prone to experiencing negative emotions. Open individuals have broad interests and prefer novelty over routine. Research has demonstrated that the Big Five model comprehensively encompasses fundamental individual differences, and other personality models can be framed and understood within the Big Five framework [2]. The field of organizational psychology has demonstrated increasing interest in the construct of job satisfaction (e.g., [3,4]). Job satisfaction encompasses cognitive, affective, and behavioral dimensions [5], and is often defined as a positive evaluative state about one’s job, expressing contentment and positive emotions towards work [6].

Numerous studies have linked job satisfaction to various workplace characteristics. In a meta-analysis, Judge et al. [7] identified a positive association between job satisfaction and job performance. Other studies have reported that satisfied employees engage in more productive work behaviors [8] and are characterized by lower rates of absenteeism [9,10]. Job satisfaction has also been negatively associated with stress [11,12,13], substance use [14,15], and positively related to marital satisfaction, as well as mental and physical health [16]. The concept of wellbeing, which encompasses satisfaction with both work and life, has been identified as critical for maintaining an effective workforce [17]. These findings highlight the importance of identifying factors that contribute to job satisfaction and why it remains a central focus of organizational psychology research [18,19].

There are several theories that can account for the link between personality traits and job satisfaction as well as the need to study the associations between personality and job satisfaction. One theory is the person–environment fit theory, which suggests that job satisfaction arises when there is a good match between one’s personality and the demands of their job [20,21,22]. In other words, individuals have a higher likelihood to be satisfied with their jobs if the work environment aligns with their personality traits. For example, an extroverted person might be more satisfied in a job that involves social interaction, while an introverted person might prefer a quieter, more solitary work environment. Another theory is the self-regulation theory, which suggests that personality traits such as Conscientiousness and emotional stability are important predictors of job satisfaction because they influence an individual’s ability to manage their own behavior and emotions in the workplace [23,24]. Conscientious people are more likely to be organized, dependable, and able to manage their workload effectively, which can lead to greater job satisfaction. Similarly, individuals who are high in emotional stability are better able to handle stress and cope with job-related challenges, which can also lead to greater job satisfaction. Finally, the social learning theory suggests that personality traits can be shaped and influenced by social and environmental factors, such as feedback from coworkers and supervisors, as well as the overall culture of the workplace [25]. In this theory, job satisfaction is seen as a product of the interaction between an individual’s personality traits and the social context in which they work.

Although how personality traits are related to job satisfaction is widely studied [4,19,24,26,27,28], it remains unclear how personality traits may relate to facets of job satisfaction, including total pay, security, work itself, and hours worked. Understanding how personality traits are related to different areas of job satisfaction can also help individuals make informed decisions about career paths and job choices that align with their personality and increase the likelihood of job satisfaction [26], given that areas of job satisfaction contribute to overall job satisfaction. For example, a person who values a work–life balance may prioritize that area of job satisfaction more highly than someone who values job security above all else, which may depend on one’s personality.

Thus, the aim of the current research is to look at how personality traits contribute to facets of job satisfaction, including total pay, security, work itself, and hours worked. This study hypothesizes that Neuroticism is negatively related to areas of job satisfaction whereas other personality traits are positively related to job satisfaction [4,19,26,27,28,29,30]. However, the patterns of these associations are diverse and may be dependent on specific dimensions of job satisfaction. 

## 2. Method

### 2.1. Data

The present investigation employed data from Wave 15 of the British Household Panel Survey (BHPS), which is a nationally representative survey of UK households that has been conducted annually since 1991 [31]. The data collection procedures for the British Household Panel Survey (BHPS) involve a multistage stratified sampling design to ensure representative household selection. The initial sample was drawn in 1991, with subsequent waves including panel members and newly added households. Recruitment combines random probability sampling and volunteer participation. The baseline survey gathers comprehensive demographic and socioeconomic information through face-to-face interviews with household members. Follow-up surveys, conducted annually or biennially, use standardized questionnaires to capture longitudinal data on various topics. Computer-assisted interviewing techniques are employed, with interviewers visiting households or conducting interviews via phone or online platforms. Ethical guidelines ensure participant confidentiality and informed consent. Quality control measures include interviewer training and supervision, data validation checks, and thorough documentation of procedures and variable definitions. The data collection procedures for the study have been approved by the University of Essex Ethics Committee, and all participants provided informed consent before taking part in the study. This dataset can be accessed via https://www.iser.essex.ac.uk/bhps (accessed on 1 April 2023).

The study included participants who met the following criteria: (a) were working for an employer (those who were self-employed were excluded), (b) were within the employable age range (16–65 years), and (c) provided complete data on areas of job satisfaction, personality, and demographics. Thus, in total 6962 participants remained in the current analysis. 

### 2.2. Measures 

#### 2.2.1. Areas of Job Satisfaction

Participants were asked to indicate how satisfied they were with areas of job satisfaction ad hoc (each begins with “I’m going to read out a list of various aspects of jobs, and after each one I’d like you to tell me from this card (E3) which number best describes how satisfied or dissatisfied you are with that particular aspect of your own present job”). Areas of job satisfaction include “The total pay, including any overtime or bonuses”, “Your job security”, “The actual work itself”, and “The hours you work”. Participants responded on a scale from 1 (“not satisfied at all”) to 7 (“completely satisfied”).

#### 2.2.2. Personality Traits

In this study, personality traits were assessed using a fifteen-item questionnaire based on the five-factor model of personality (BFI-S; [32]). The questionnaire consisted of three questions for each of the five personality dimensions, which were scored using a Likert scale ranging from 1 (“does not apply to me”) to 7 (“applies to me perfectly”). The mean score averaged across items was used to represent each trait. Internal consistency was evaluated using Cronbach’s alpha, and the values for each trait were: Neuroticism = 0.69, Openness = 0.66, Extraversion = 0.60, Agreeableness = 0.57, and Conscientiousness = 0.54). Previous studies have also demonstrated the reliability of this short questionnaire through test–retest correlations as well as its convergent and discriminant validity [33,34,35].

#### 2.2.3. Control Variables

Control variables included age, sex, education, occupation, marital status, annual income, hours per week, and overtime per week. Please refer to Table 1. for the coding of these variables. 

### 2.3. Statistical Analysis

Due to the skewness of the outcome variables, we decided to run four ordinal logistic regression models to analyze the associations between personality traits and areas of life satisfaction. Specifically, the predictors in these models consisted of personality traits, including Agreeableness, Extraversion, Conscientiousness, Openness, and Neuroticism; and the control variables including age, sex, education, occupation, marital status, annual income, hours per week, and overtime per week. While the predictors were the same for each logistic regression model, the outcome variables for these logistic regression models were job satisfaction for total pay; job satisfaction for security; job satisfaction for work itself; and job satisfaction for hours worked, respectively. We carried out all analyses using MATLAB 2018a. 

## 3. Results

Descriptive statistics are shown in Table 1. Results indicated that Neuroticism (OR = 0.82, 95% CI [0.78, 0.86], *p* <0.001) and Extraversion (OR = 0.95, 95% CI [0.90, 0.99], *p* < 0.05) have negative associations with job satisfaction for total pay, whereas Agreeableness (OR = 1.08, 95% CI [1.03, 1.14], *p* < 0.01) and Conscientiousness (OR = 1.07, 95% CI [1.02, 1.12], *p* < 0.01) were positively associated with job satisfaction for total pay. Openness (OR = 0.99, 95% CI [0.94, 1.04], *p* = 0.68) had no association with job satisfaction for total pay (Table 2).

Similarly, higher levels of Neuroticism were related to lower levels of job satisfaction for security (OR = 0.82, *p* < 0.01, 95% CI [0.78, 0.86]). In contrast, higher levels of Agreeableness (OR = 1.13, *p* < 0.01, 95% CI [1.07, 1.18]) and Conscientiousness (OR = 1.16, *p* < 0.01, 95% CI [1.11, 1.22]) were associated with higher levels of job satisfaction for security. Openness and Extraversion were not significantly associated with job satisfaction for security (*p* > 0.05; Table 2).

Moreover, higher levels of Agreeableness (OR = 1.17, 95% CI [1.11, 1.23]), Conscientiousness (OR = 1.19, 95% CI [1.14, 1.26]), and lower levels of Neuroticism (OR = 0.78, 95% CI [0.74, 0.82]) were associated with higher odds of job satisfaction for work itself. Openness (OR = 1.04, 95% CI [0.99, 1.10]) and Extraversion (OR = 1.03, 95% CI [0.98, 1.08]) were not significantly related to job satisfaction for work itself (Table 2).

Finally, Neuroticism had a negative association with job satisfaction for hours worked (OR = 0.82, *p* < 0.001, 95% CI [0.79, 0.86]). Agreeableness (OR = 1.17, *p* < 0.001, 95% CI [1.11, 1.23]) and Conscientiousness (OR = 1.10, *p* < 0.001, 95% CI [1.05, 1.16]) were positively associated with job satisfaction for hours worked. Openness (OR = 1.03, *p* = 0.30, 95% CI [0.98, 1.08]) and Extraversion (OR = 0.97, *p* = 0.14, 95% CI [0.92, 1.01]) were not significantly associated with job satisfaction for hours worked (Table 2).

## 4. Discussion

Our aim was to investigate the associations between the Big Five and areas of job satisfaction including pay, work itself, security, and hours worked. Results revealed that Neuroticism is a consistent negative predictor of all aspects of job satisfaction, whereas Agreeableness and Conscientiousness consistently have a positive association with aspects of job satisfaction. Extraversion had a weak negative association with satisfaction with total pay. These results may indicate that if one aspect of personality traits has an association with a certain aspect of job satisfaction, then it perhaps would be similarly associated with other aspects of job satisfaction. 

Neuroticism represents a personality disposition marked by a proclivity to encounter unfavorable affective states, including anxiety, fear, and worry. The negative associations found between Neuroticism and areas of job satisfaction seemed to be consistent in studies regarding the association between Neuroticism and overall job satisfaction [4,19,26,28,29,36]. One possible explanation for this negative relationship is that neurotic individuals have a more negative perception of their work environment, leading to lower levels of job satisfaction. Additionally, people high in Neuroticism may experience more stress and anxiety in response to work-related challenges, which can also impact job satisfaction. 

Agreeableness is the trait that inclines individuals to be cooperative, empathetic, and compassionate toward others. Empirical evidence consistently supports that individuals scoring high in Agreeableness are more likely to express higher levels of job satisfaction (e.g., [4,19,26,28,29,36]) and achieve satisfaction in comparison to those who score low in Agreeableness. One possible explanation for this relationship is that agreeable individuals tend to have better interpersonal relationships with coworkers and supervisors, leading to more positive work experiences and greater job satisfaction. Agreeable individuals may tend to engage in pro-social behaviors such as helping others and resolving conflicts, which can lead to a more positive work environment. Another explanation is that agreeable individuals may be more likely to perceive their work as meaningful and fulfilling, leading to greater job satisfaction. Agreeable individuals may derive satisfaction from contributing to the well-being of others or making a positive impact on their work environment.

Conscientiousness is a personality trait characterized by being responsible, reliable, and organized, among other attributes. Research has found that individuals high in Conscientiousness tend to experience higher levels of job satisfaction [4,19,26,28,29,36]. This is likely because conscientious individuals tend to approach their work in a diligent and committed manner, which can lead to feelings of accomplishment and satisfaction when tasks are completed successfully [37]. Additionally, there were links between Conscientiousness and various job-related behaviors, such as task performance, job performance, and organizational citizenship behaviors [27,38], which can also contribute to higher levels of job satisfaction.

Finally, Extraversion had a weak negative association with satisfaction with total pay. Extraversion is a personality trait characterized by sociability, assertiveness, and positive emotions. Research has found that Extraversion is negatively associated with job satisfaction [19,26,28,29,36]. One explanation for this finding is that highly extraverted individuals are more likely to be motivated by social rewards such as recognition, social status, and social interactions rather than material rewards such as pay [24]. As a result, they may prioritize social rewards when evaluating their job satisfaction. Another explanation is that highly extraverted individuals may have higher expectations for their pay, due to their self-confidence and assertiveness [24,39]. When these expectations are not met, they may experience lower satisfaction with their pay.

This study controlled several factors, including income, hours worked, and overtime. However, some personality traits were still significant after controlling for these factors, which may indicate that job satisfaction cannot be fully explained by their actual conditions [40], but may also be determined by individual differences in terms of individual perception. These findings are consistent with previous findings that observe that the actual job condition has rather small associations with job satisfaction [40]. 

Several shortcomings of this research should be noted. First, aspects of job satisfaction are not limited to satisfaction with pay, security, work itself, and hours worked, but include other aspects such as working environment, social relationships with colleagues, and career development. Thus, it would be important for future studies to assess how the Big Five may be associated with other aspects of job satisfaction. Second, the use of self-reported data has limitations and challenges in research. Self-report assessments are subject to biases, including social desirability bias, memory bias, and acquiescence bias. Moreover, self-reported data might not always be accurate or reliable as it is influenced by the participant’s subjective perception and interpretation of the questions. Therefore, future studies should use multiple methods of data collection to triangulate findings and reduce potential biases. Third, although this study controlled for occupation, the relationships between the Big Five and areas of job satisfaction may depend on occupation [19]. Future research should test if occupation moderates the connections between the Big Five and areas of job satisfaction. Finally, some effects were near the threshold of 0.05, and the data were quite old; thus, interpreting these results must be accompanied with caution. 

In conclusion, this study provides evidence that personality traits are related to various aspects of job satisfaction. Neuroticism is a consistent negative predictor of job satisfaction, while Agreeableness and Conscientiousness consistently have a positive association with job satisfaction. Extraversion had a weak negative association with satisfaction with pay. The patterns of these associations seem to be mostly unitary rather than diverse. Our findings suggest that personality may play a crucial role in shaping job satisfaction. Organization could use results for selecting employees. For instance, they may be interested in hiring employees with low Neuroticism scores but high Agreeableness scores, as they tend to be more satisfied with aspects of their job, which then may lead to better job performance [36]. 

## Figures and Tables

**Table 1:** The descriptive statistics for variables of interests in this study. Mean and standard deviation (SD) are reported for continuous variables, whereas count and percentage were reported for categorical variables.

Variables		Mean	SD
Age		38.20	12.50
Annual income		19,388.75	19,756.23
Hours per week		32.94	11.36
Overtime per week		3.24	5.60
Neuroticism		3.67	1.25
Agreeableness		4.60	1.11
Openness		5.44	0.95
Conscientiousness		5.38	0.98
Extraversion		4.57	1.09
	Value	Count	Percentage
Sex	Male	3256	46.77%
	Female	3706	53.23%
Education	Higher Degree	276	3.96%
	First Degree	1082	15.54%
	Teaching QF	124	1.78%
	Other Higher QF	2178	31.28%
	Nursing QF	51	0.73%
	GCE A Levels	1052	15.11%
	GCE O Levels or Equivalent	1256	18.04%
	Commercial QF, No O	101	1.45%
	CSE Grade 2–5, Scot G	227	3.26%
	Apprenticeship	42	0.60%
	Other QF	32	0.46%
	No QF	513	7.37%
	Still At School, No Q	28	0.40%
Marital status	Married	3645	52.36%
	Separated	179	2.57%
	Divorced	575	8.26%
	Widowed	88	1.26%
	Never married	2475	35.55%
Job satisfaction: total pay	1 (“not satisfied at all”)	659	9.47%
	2	2687	38.60%
	3	1789	25.70%
	4	524	7.53%
	5	830	11.92%
	6	278	3.99%
	7 (“completely satisfied”)	195	2.80%
Job satisfaction: security	1 (“not satisfied at all”)	1746	25.08%
	2	2789	40.06%
	3	1233	17.71%
	4	491	7.05%
	5	413	5.93%
	6	154	2.21%
	7 (“completely satisfied”)	136	1.95%
Job satisfaction: work itself	1 (“not satisfied at all”)	1117	16.04%
	2	3187	45.78%
	3	1502	21.57%
	4	471	6.77%
	5	417	5.99%
	6	171	2.46%
	7 (“completely satisfied”)	97	1.39%
Job satisfaction: hours worked	1 (“not satisfied at all”)	1039	14.92%
	2	2746	39.44%
	3	1625	23.34%
	4	639	9.18%
	5	638	9.16%
	6	190	2.73%
	7 (“completely satisfied”)	85	1.22%

**Table 2:** The results of ordinal logistic regressions using control variables and personality traits as predictors to predict aspects of job satisfaction including A. job satisfaction for total pay, B. job satisfaction for security, C. job satisfaction for work itself, and D. job satisfaction for hours worked.

A. Job satisfaction for total pay.			
**Variables**	**OR**	***p***	**95% C.I.**
Age	1.26	<0.001	[1.14, 1.40]
Sex	1.04	<0.001	[1.02, 1.06]
Occupation	1.00	<0.001	[1.00, 1.00]
Marital status	0.96	<0.05	[0.94, 0.99]
Annual income	1.00	<0.001	[1.00, 1.00]
Hours per week	0.98	<0.001	[0.98, 0.98]
Overtime per week	1.00	0.44	[1.00, 1.01]
Neuroticism	0.82	<0.001	[0.78, 0.86]
Agreeableness	1.08	<0.01	[1.03, 1.14]
Openness	0.99	0.68	[0.94, 1.04]
Conscientiousness	1.07	<0.01	[1.02, 1.12]
Extraversion	0.95	<0.05	[0.90, 0.99]
B. Job satisfaction for security.			
**Variables**	**OR**	***p***	**95% C.I.**
Age	1.47	<0.001	[1.32, 1.62]
Sex	1.02	<0.05	[1.00, 1.04]
Occupation	1.00	<0.05	[1.00, 1.00]
Marital status	1.01	0.67	[0.98, 1.04]
Annual income	1.00	0.65	[1.00, 1.00]
Hours per week	1.00	0.60	[0.99, 1.00]
Overtime per week	1.01	<0.05	[1.00, 1.02]
Neuroticism	0.82	<0.01	[0.78, 0.86]
Agreeableness	1.13	<0.01	[1.07, 1.18]
Openness	1.01	0.616	[0.97, 1.06]
Conscientiousness	1.16	<0.01	[1.11, 1.22]
Extraversion	0.98	0.50	[0.94, 1.03]
C. Job satisfaction for work itself.			
**Variables**	**OR**	***p***	**95% C.I.**
Age	1.32	<0.001	[1.19, 1.46]
Sex	1.05	<0.001	[1.03, 1.07]
Occupation	1.00	<0.001	[1.00, 1.00]
Marital status	0.99	0.33	[0.96, 1.01]
Hours per week	1.00	0.26	[1.00, 1.00]
Overtime per week	0.99	<0.01	[0.99, 1.00]
Annual income	1.01	<0.05	[1.00, 1.02]
Neuroticism	0.78	<0.001	[0.74, 0.82]
Agreeableness	1.17	<0.001	[1.11, 1.23]
Openness	1.04	0.08	[0.99, 1.10]
Conscientiousness	1.19	<0.001	[1.14, 1.26]
Extraversion	1.03	0.21	[0.98, 1.08]
D. Job satisfaction for hours worked.			
**Variables**	**OR**	***p***	**95% C.I.**
Age	1.11	<0.05	[1.00, 1.23]
Sex	1.05	<0.001	[1.03, 1.07]
Occupation	1.00	<0.05	[1.00, 1.00]
Marital status	1.00	0.81	[0.97, 1.03]
Hours per week	1.00	0.72	[1.00, 1.00]
Overtime per week	0.97	<0.001	[0.97, 0.97]
Annual income	0.95	<0.001	[0.94, 0.95]
Neuroticism	0.82	<0.001	[0.79, 0.86]
Agreeableness	1.17	<0.001	[1.11, 1.23]
Openness	1.03	0.30	[0.98, 1.08]
Conscientiousness	1.10	<0.001	[1.05, 1.16]
Extraversion	0.97	0.14	[0.92, 1.01]

## Data Availability

Publicly available datasets were analyzed in this study. These data can be found here: https://www.iser.essex.ac.uk/bhps (accessed on 1 April 2023).
